# Small Airways Involvement in Patients with Rheumatoid Arthritis

**DOI:** 10.5539/gjhs.v5n2p166

**Published:** 2012-12-26

**Authors:** Mohammad Ali Zohal, Zohreh Yazdi, Ali Rassi Ghaemi, Mahnaz Abbasi

**Affiliations:** 1 Metabolic Diseases Research Center, Qazvin University of Medical Sciences, Qazvin, Iran; 2 Occupational Medicine Specialist, Qazvin University of Medical Sciences, Qazvin, Iran; 3 General Practitioner, Qazvin, Iran

**Keywords:** rheumatoid arthritis, obstructive lung diseases, spirometry

## Abstract

**Objectives:**

One of the common causes of morbidity in patients with RA is pulmonary involvement. Some studies have shown that the possible abnormal results of pulmonary function tests in rheumatoid disease are higher than usual. We aimed to evaluate the prevalence of spirometric abnormalities in patients with RA.

**Materials & Methods:**

This case-control study was conducted on 99 patients with RA who referred to a rheumatology clinic in Qazvin, northwest Iran. Sixty five age- and sex-matched healthy controls were recruited as well. History taking, physical examination, laboratory tests and spirometry were performed for the participants. RA severity was assessed according to Disease Activity Score 28 (DAS28). The data were processed using SPSS software version 16. Chi square and student's t test and multiple logistic regressions were used as appropriated.

**Results:**

The mean (±SD) age of the patients was 46 (±10.5) years. The mean (±SD) duration of disease was 4.8 (±5.4) years, and the mean (±SD) DAS_28_ was 2.5 (±1.1). Dyspnea was the most common respiratory complaint (6.1%). Three (3%) patients had mild restrictive, 2 (2%) patients mild obstructive, and one (1%) patient moderate obstructive diseases. In the control group, only one participant had mild restrictive pulmonary disease (P<0.05). A significant decrease of FEF25 [OR=3.2; 95%CI (1.9-4.5)], FEF50 [OR=2.5; 95% CI (1.7-3.1)], FEF75 [OR=2.3; 95% CI (1.4-2.7)] and FEF25-75 [OR=2.7; 95% CI (1.7-3.5)] was observed in patients compared with the control group. We found no correlation between the patients’ age, duration and severity of the disease, and laboratory tests with spirometric indices.

**Conclusion:**

It is recommended that patients with RA be visited on a regular basis and PFT be done for them for the early diagnosis of pulmonary involvement.

## 1. Introduction

Rheumatoid arthritis (RA) is a chronic systemic inflammatory disease with an unknown origin. It can have various systemic presentations; however the main characteristic of the diseases is symmetrical inflammatory synovitis, which affects the peripheral joints. The lung can be involved frequently in rheumatological diseases. This involvement can be caused by different etiologies such as drug toxicity, infection, or specific manifestations of the immune process (Raniga et al., 2006; Gabbay et al., 1997).

One of the most common causes of morbidity and mortality in patients with RA is pulmonary involvement, which is the second leading cause of death after the infection (Raniga et al., 2006). It is has been approved that interstitial lung disease complicates patients with RA. However, various studies on small airway involvement in such patients yielded controversial results (Avnon et al., 2009; Perez et al., 1998; Cortet et al., 1997; Hassan et al., 1994; Geddes et al., 1979). In some studies, obstructive lung disease has been documented in 60% of patients. Different etiologies have been proposed for this high prevalence, including concurrent use of cigarettes, coexistence of rheumatoid diseases such as Sjögren syndrome, bronchiectasis, and side effects of anti-rheumatoid drugs (Perez et al., 1998; Saag et al., 1996). Some studies showed airways dysfunction in 38-65% of patients (Perez et al., 1998). In one study, the forced expiratory volume in 1s (FEV1) was decreased (Geddes et al., 1979). In another study carbon monoxide diffusion capacity (DLCO) was examined, which showed an increase in the capacity release (Instow et al., 1994).

Some studies have related the airway disorder and mean maximum mid-expiratory flow rate (MEFR) reduction in such patients to smoking, and not to the rheumatoid process (Banks et al., 1992). Furthermore, some studies have shown that the possible abnormal results of pulmonary function tests in rheumatoid disease are higher than usual and based on it, they have suggested that performing periodical spirometry in these patients could be useful (Avnon et al., 2009). Considering the existing controversies, we aimed to evaluate the prevalence of spirometric abnormalities in patients with RA.

## 2. Methods

This case-control study was conducted on patients with RA whose disease were diagnosed and confirmed by a single rheumatologist according to the revised version of the criteria released by the American College of Rheumatology in 1987 (Arnett et al., 1988). The patients were referred from May to October 2010 to the Rheumatology Clinic of Qazvin Bu-Ali Hospital, Qazvin, northwest Iran. All known cases and newly diagnosed outpatients who had visited the rheumatology clinic consecutively were enrolled in the study. All the patients were consuming at least one Disease Modifying Antirheumatic Drugs (DMARDs). The control group was selected among the patients’ fellows or individuals without RA. The patients in the control group were assessed by a rheumatologist to rule out RA. In both groups, the patients with a history of smoking, prior pulmonary disease and previous history of occupational dust or fume exposure were excluded.

Sample size calculation was performed on the basis of previous studies with 40% increase at the spirometric abnormalities in RA patients compared with normal people. Sample size was calculated with Epi info 6 software with α= 0.05 and β=0.2. Therefore, 100 patients with RA and 50 normal controls were calculated. Finally, 99 patients and 65 healthy controls were assessed in the study (Perez et al., 1998).

The study protocol was approved by the Vice Chancellorship for Research at Qazvin University of Medical Sciences. Written informed consents were obtained from the participants.

In order to record the patients’ information, the patients histories were taken and complete physical examinations were performed. The disease activity was investigated by the DAS_28_ criteria, as follows:

DAS28 = 0.56 * sort (number of tender joints 0-28) + 0.28 * sort (number of swollen joints 0- 28) + 0.70 * ln (ESR,mm/hr) + 0.014 * Vas general health patients (mm)

Decrease in patients’ functionality was evaluated based on the used functional class in rheumatoid arthritis (Hochberg et al., 1992). All the patients were asked about the followings: employment, history of previous diseases, pulmonary symptoms such as cough and phlegm, dyspnea, chest pain, and wheezing. The laboratory tests requested for the patients included: assessment of rheumatoid factor (RF), C-reactive protein (CRP), erythrocyte sedimentation rate (ESR), anti-cyclic citrullinated peptide (Anti-CCP), and anti-nuclear antibody test (ANA). Then, the participants were referred to the pulmonologist for spirometry and interpretation of the related findings. The spirometry was performed according to the American Thoracic Society (ATS) criteria (Miller et al., 2005). The evaluated indices in pulmonary function test included forced expiratory flow (FEF_25%-75%_)_,_ FEF_75_, FEF_50_, FEF_25_, forced vital capacity (FVC), forced expiratory volume (FEV_1_), FEV_1_/FVC and peak expiratory flow (PEF). Based on the ATS criteria, those who had an FEV_1_/FVC of less than 70%, were identified as having obstructive disease; its severity was determined according to FEV_1_ decline. The patients with normal FEV_1_/FVC and decreased FVC (<80%) were diagnosed as having restrictive disease; its severity was determined by the decrease in FVC.

Data were analyzed using SPSS software, version 16. The group data was introduced with the mean and standard deviation. Quantitative and qualitative data were analyzed using student's *t* Chi-square tests, respectively. Logistic regression was used to calculate the odds ratio (OR) and confidence interval of the variables. P values less than 0.05 were considered as statistically significant.

## 3. Results

Overall, 99 patients and 65 health controls were studied. The mean (±SD) age of the studied patients in the case group was 46 (±10.5) years (range: 19-73). 89 (89.9%) patients were women and 10 (10.1%) were men. The control group consisted of 10 (15.4%) men and 55 (84.6%) women with a mean (±SD) age of 45.6 (±7.9) years (range: 35-65,). The difference in the mean ages of the patients in the case and control groups was not statistically significant (P=0.5). Also, there was no difference in sex distribution between these 99 patients and the 65 controls. None of the patients and controls smoked.

The mean (±SD) duration of RA was 4.8 (±5.4) years. Of the 99 patients with RA, 81 (81.8%) patients were receiving methotrexate, 97 (98%) hydroxychloroquine, 3 (3%) sulfasalazine, and 1 (%1) was receiving non-steroidal anti inflammatory drug (NSAID). All the patients were receiving prednisolone.

The mean (±SD) age of the patients at the onset of the disease was 41 (±11.3) years (range: 8-67). There were only six (1.6%) patients who had pulmonary complaints. Three (3%) patients had sputum and 5 (5.1%) patients had cough. All these six patients complained from dyspnea along with other symptoms, which was the most frequent reported pulmonary symptom.

The mean (±SD) ESR in the patients was 17.5 (±11.6) (range: 2-60). 87 (87.9%) patients were in functional class I, 11 (11.1%) in class II, and one (1%) in class III. No patient was in the functional class IV. The mean (±SD) DAS_28_ was 2.5 (±1.1) in the case group, which shows that many patients had DAS_28_ <2.6 which was the characteristic of the recovery phase of the disease.

Table 1 shows the results of laboratory tests in the case group.

**Table 1:** Results of laboratory tests in the patients’ group

	Positive	Negative
**RF**	24 (24.2%)	75 (75.7%)
**Anti CCP**	11 (11.1%)	88 (88.9%)
**ANA**	3 (3%)	96 (97%)
**CRP**	47 (47.5%)	52 (52.5%)

Table 2 shows the comparison of the spirometric variables between both groups of patients. Based on the multiple logistic regression results, the values for FEF_25_ [OR=3.2; 95%CI (1.9-4.5)], FEF_50_ [OR=2.5; 95% CI (1.7-3.1)], FEF_75_ [OR=2.3; 95% CI (1.4-2.7)], and FEF_25-75_ [OR=2.7; 95% CI (1.7-3.5)] were significantly lower in the case group compared with the control group.

**Table 2:** Mean of spirometry parameters in the case and control groups

	Patients (n=99)	Controls (n=65)	OR (95% CI)	P-value
FVC%	97/6±11/9	98/4±11/8	1.21 (0.97-1.47)	0.15
FEV_1_%	98/7±13/5	98/8±10/8	1.65 (0.84-2.1)	0.07
FEV_1_/FVC%	85±6/8	85/6±3/8	-0.93 (0.79-1.13)	0.41
PEF	99/3±22/3	99/7±18	1.5 (0.86-1.43)	0.09
FEF_25_	84/6±33/6	100/5±17/4	3.2 (1.9-4.5)	0.002
FEF_50_	97/9±25/1	113/4±24/3	2.5 (1.7-3.1)	0.01
FEF_75_	102/8±22/2	112/9±33	2.3 (1.4-2.7)	0.007
FEF_25-75_	94/2±27/7	111/9±22/2	2.7 (1.7-3.5)	0.03
VC	94/5±10/7	95/1±12/3	2.1 (0.7-2.8)	0.21

Small airway involvement in patients with RA was confirmed. In pulmonary function studies, 93 (93.9%) patients were normal, 3 (3%) had mild restrictive, 2 (2%) mild obstructive, and one (1%) moderate obstructive diseases. In the control group, 64 (98.5%) individuals had normal tests and only one (1.5%) participant had mild restrictive pulmonary disease (P<0.05).

We found no correlation between the patients’ age, duration and severity of the disease, and laboratory tests with spirometric indices.

## 4. Discussion

The mean values of FEF_25-75_, FEF_75_, FEF_50,_ and FEF_25_ were significantly lower in the case group compared with the control group. This fact indicated small airway involvement in patients with RA. Many studies have been conducted on the prevalence of pulmonary disorders in the patients with RA. In such studies, various pulmonary tests including spirometry and high resolution CT have been performed for the assessment of the existence and the type of lung involvement. Based on the results of these studies the incidence of lung involvement in patients with RA was reported to be 38-65% (Perez et al., 1998). Different reports exist regarding the type of pulmonary involvement. Some studies have shown that the most common type of involvement was restrictive disease and the failure in gas transfer (Fuld et al., 2003). The results of other studies indicated obstructive pulmonary diseases--especially in the small airways (Mori et al., 2011). Researchers who used HRCT have reported bronchiectasis as the most commonly observed lung involvement is such patients (Avnon et al., 2009; Geddes et al., 1979; Instow et al., 1994). The observed differences in the results of various studies could be somehow related to the different study design and the difference in diagnostic tools used in various studies. Fuld and colleagues (2003) reported that PFT abnormalities in patients with RA were higher than the control group, which is consistent with our findings. In another study conducted in 2009 the involvement of small airways was the most common PFT abnormality in patients with RA. In that study, the impairment in the PFT was more in patients with RA than other patients (Avnon et al., 2009). Ayhan and colleagues reported similar results in 2006. The difference between our findings and the above studies was the higher percentage of PFT abnormalities in the previous studies. As it was noted, the incidence of pulmonary dysfunction was reported to be 38-65% in previous studies (Perez et al., 1998), which was much higher than the reported rates in our study. This difference could be due to lower disease severity, less disease duration, and the lower age of the patients in our study.

There was no correlation between age and PFT abnormalities in our study. This was in contrast with the report of Jamshidi and colleagues (2004) who found correlations between age and PFT results. In our study, there was no significant correlation between the duration and severity of the disease (based on DAS_28_) with PFT abnormalities. There was no significant correlation between laboratory tests (such as ANA, CRP, ESR and Anti-CCP), with PFT abnormalities either. Similar results were derived from the studies of Jamshidi and co-workers (2004) and Ayhan and colleagues (2006). In the latter study, no relationship was found between disease duration and activity with PFT abnormalities in patients with RA (Ayhan-Ardic et al., 2006).

In our study, there was no significant correlation between the prevalence of PFT abnormalities with sex, which is in accordance with Jamshidi (2004) and Fuji (1993) studies, who reported similar findings. However, Gabbay and colleagues (1997) showed that the risk of lung involvement was more in men. In our study, there was no significant relationship between drug type and duration of receiving drugs with the PFT disorders. In our study, unlike the study of Jamshidi and co-workers (2004) there was no significant correlation between pulmonary symptoms (such as dyspnea) and PFT disorders.

Our study had some limitations. Firstly, the patients in our study were selected from a university hospital, which could create bias in selecting patients with more severe involvement compared with patients at the community level, although all our patients were recruited from the hospital's outpatient clinic. The second limitation of our study was that we could not used additional pulmonary tests (including body-box) to evaluate the restrictive lung diseases more accurately. This test was not available in our center.

## 5. Conclusion

Pulmonary involvement can be seen in patients with RA. While pulmonary involvement has a significant role in the mortality and morbidity of such affected patients, since the involvement progresses gradually with few signs and symptoms, most patients may not have obvious complaints. Therefore, it is recommended that patients with RA be visited on a regular basis and PFT be done for them for the early diagnosis of pulmonary involvement.
